# Root bark of *Ulmus davidiana var*. *japonica* restrains acute alcohol-induced hepatic steatosis onset in mice by inhibiting ROS accumulation

**DOI:** 10.1371/journal.pone.0188381

**Published:** 2017-11-27

**Authors:** Jeong Hoon Pan, Yejin Lim, Jun Ho Kim, Wan Heo, Ki Yong Lee, Hye Ji Shin, Jae Kyeom Kim, Jin Hyup Lee, Young Jun Kim

**Affiliations:** 1 Department of Food and Biotechnology, Korea University, Sejong, Republic of Korea; 2 School of Human Environmental Sciences, University of Arkansas, Fayetteville, Arkansas, United States of America; 3 College of Pharmacy, Korea University, Sejong, Republic of Korea; Medizinische Fakultat der RWTH Aachen, GERMANY

## Abstract

Alcohol-induced hepatic steatosis and inflammation are key drivers of alcohol-induced liver injury, mainly caused by oxidative stress. The roots bark of *Ulmus davidiana var*. *japonica* is well known for its substantial antioxidative and antitumorigenic potency. In this study, we examined whether this plant can ameliorate alcohol-induced liver injuries characterized by hepatic steatosis and inflammation through its antioxidative activity. C57BL/6J mice were treated with the root bark extract of *Ulmus davidiana var*. *japonica* (RUE; 100 mg of extract/kg bodyweight; oral gavage) and alcohol (1 g/kg of bodyweight; oral gavage) for 5 days. Markers of acute alcohol-induced hepatic steatosis were determined and putative molecular mechanisms responsible for the protection of RUE were investigated. RUE noticeably protected against alcohol-induced hepatic steatosis and inflammation. Reactive oxygen species (ROS), over-produced by alcohol, negatively orchestrated various signaling pathways involved in the lipid metabolism and inflammation. These pathways were restored through the ROS scavenging activity of RUE in the liver. In particular, the expression of lipogenic genes (e.g., SREBP-1, ACC, and FAS) and inflammatory cytokines (e.g., IL-1β, and NF-κB p65) significantly decreased with RUE treatment. Conversely, the expression of fatty acid oxidation-related genes (e.g., SIRT1, AMPKα, and PGC1α) were increased in mice treated with RUE. Thus, the results indicate that RUE counteracts and thus attenuates alcoholic hepatic steatosis onset in mice, possibly by suppressing ROS-mediated steatosis and inflammation.

## Introduction

Alcohol-induced liver injury is a complex pathological process that includes a wide spectrum of hepatic lesions, and alcoholic liver injury is one of major causes of death from liver diseases worldwide [1]. Hepatic steatosis is a representative status of liver disease that can be induced by alcohol consumption. In this regards, various studies have suggested that alcohol consumption induces hepatic lipogenesis [2, 3] and suppresses fatty acid oxidation in liver [4, 5] in parallel with hepatic inflammation [6]. These disruptions result from excessive generation of reactive oxygen species (ROS) [7–9]. In particular, alcohol metabolites deplete glutathione (GSH) levels and lead to ROS-mediated liver damages associated with hepatic steatosis and inflammation [10, 11]. Therefore, the supplementation of a synthetic or natural antioxidant that scavenges the harmful ROS may be a good preventive strategy for alcohol-induced hepatic steatosis.

*Ulmus davidiana var*. *japonica* is widely distributed in Asian countries. The bioactive constituents in the root bark extract of *Ulmus davidiana var*. *japonica* (RUE) have been reported to have potent antioxidative [12, 13] and antitumorigenic [14–16] activities. Naturally occurring constituents have been demonstrated to improve alcohol-induced hepatic steatosis by enhancing hepatic antioxidation and anti-inflammation activities [17]. However, to the best of our knowledge, no previous study has attempted to examine the effects of RUE on alcohol-induced hepatic steatosis. In this study, we hypothesized that RUE may have beneficial effects in alcohol-induced hepatic steatosis owing to its potent antioxidative activity.

In this study, mice were treated with RUE following alcohol administration. The protective effects of RUE against the alcoholic hepatic steatosis were assessed by analyzing the level of lipid accumulation, inflammation, and apoptosis of the liver tissue in mice. In particular, the mechanisms and signaling pathways responsible for the injury (lipogenesis, fatty acid oxidation, MAPK-NF-κB-mediated inflammatory signaling pathway, and MAPK-p53-mediated apoptotic pathway) were comprehensively investigated. Finally, the levels of ROS, oxidative damage, and redox status were measured in the tissue to verify whether the beneficial effects of RUE against alcohol-induced hepatic steatosis were associated with its antioxidant capacity.

## Materials and methods

### Preparation of RUE

The root bark of *Ulmus davidiana var*. *japonica* was purchased a local medical herb market (Seoul, Republic of Korea). After being obtained, the root bark of *Ulmus davidiana var*. *japonica* was identified and specimen voucher was issued by the College of Pharmacy, Korea University (KUSCP-08). Fifty gram of ground roots bark of *Ulmus davidiana var*. *japonica* was submerged in 1 L of 75% ethanol and the sample was extracted by shaking for 24 h at room temperature (25 ± 2°C). The extract was filtered through Whatman No. 41 filter paper (Whatman Int. Ltd., Maidstone, UK) and freeze-dried using a programmable freeze dryer (Ilshin Lab Co., Yangju, Republic of Korea).

### Animals and experimental design

The care and treatment of experimental animals conformed to a protocol approved by the Institutional Animal Care and Use Committee of Korea University (Seoul, Republic of Korea). A total of 18 C57BL/6J mice (8-week-old) were purchased from Central Lab. Animal Inc. (Seoul, Republic of Korea) and housed in individual cages in a windowless room with a 12 h-light–dark cycle at a constant temperature of 25 ± 2°C and humidity of 50 ± 5%. In order to assess animal health and well-being, body weight, food intake, and the physical behavior were carefully monitored every day before and after sample administration. Mice were fed standard ingredient chow (Central Lab. Animal Inc.). Mice were assigned to negative control group (N-Con, normal saline, n = 6), positive control group (Con, 1g/kg of body weight of alcohol, n = 6), and RUE group (100 mg/kg of body weight, n = 6). The normal saline or RUE was administrated by oral gavage 30 min before alcohol administration once daily for 5 days. The mice were eventually euthanized by exsanguination via cardiac puncture while under anesthesia with i.p. injection of Avertin (240 mg/kg of body weight; 2, 2, 2-tribromoethanol, Sigma-Aldrich, St. Louis, MO). Liver tissue samples were collected and the tissues were stored at -80°C or fixed in 4% paraformalin solution until analyzed.

### Measurement of TG in serum and liver

Blood samples were collected by cardiac puncture and centrifuged at 2,000 × g for 20 min at 4°C. The serum level of TG was measured using the Serum Triglyceride Determination kit (Sigma-Aldrich). For hepatic TG measurement, tissues were saponified and neutralized as previously described [18].

### Measurement of serum aspartate aminotransferase and alanine aminotransferase levels

The levels of aspartate aminotransferase (AST) and alanine aminotransferase (ALT) in serum were measured using Cobas c-111 biochemical analyzer (Roche, Basel, Switzerland). The experiment was performed according to the manufacturer’s instruction.

### Measurement of hepatic lipid accumulation

Frozen sections of liver (5 μm) were stained with BODIPY (Molecular Probes, Eugene, OR, USA). Nuclei of the tissue were localized by counterstaining with 4',6-diamidino-2-phenylindole (DAPI), and the stained liver sections were visualized using a fluorescence microscopy (Carl Zeiss AG, Oberkochen, Germany).

### Apoptosis assay

Terminal deoxynucleotidyl transferase-mediated dUTP nick-end labeling (TUNEL) method was applied to determine hepatic apoptosis using the fluorescein in situ cell death detection kit (In Situ Cell Death Detection Kit, Roche Diagnostics, Mannheim, Germany). Nuclei of the tissue were localized by counterstaining with DAPI and visualized by fluorescence microscopy. The fluorescein isothiocyanate-labeled tissue and cells undergoing apoptosis were recognized by their green fluorescent nuclei.

### Inflammatory cytokines array

The Mouse Cytokine Antibody Array kit, Panel A (ARY006; R&D Systems, Minneapolis, MN, USA) was utilized to screen alterations in various inflammatory cytokines in the serum. The experiment was performed according to the manufacturer’s instructions. Equal volumes of serum were collected from individual animal and the serum achieved in same group were pooled in one tube. The serum mixtures were added to the pre-coated membranes of the kit. The dot blot membranes were analyzed using *ImageJ* software (Bethesda, MD, USA).

### Immunohistochemistry and immunofluorescence

For immunohistochemistry, liver sections were fixed in 4% paraformaldehyde (PFA) and blocked with 0.3% H_2_O_2_ for 10 min. Tissue sections were washed with phosphate-buffered saline (PBS) and permeabilized with 0.2% Triton X-100 in PBS for 10 min. Blocked tissue sections were probed with primary antibodies (1:100 dilution in 0.5% normal goat serum) at 4°C overnight. Horseradish peroxidase-conjugated anti-rabbit or anti-mouse IgG secondary antibody was used. After washing, sections were counterstained with Gill No. 3 hematoxylin for 3 min and washed with tap water for 10 min. In addition to immunohistochemistry, immunofluorescence was performed. Briefly, liver sections were fixed in 4% PFA (v/v) and permeabilized with 0.2% Triton X-100 in PBS for 10 min. Sections were blocked with 10% normal goat serum in PBS for 1 h at room temperature, followed by incubation with primary antibodies (1:200 dilution in 0.5% normal goat serum) at 4°C overnight. Three consecutive 5-min washes with PBS were followed by sequential incubation with fluorophore-conjugated secondary antibodies (1:200 dilution in photobleaching media) at room temperature for 30 min. The nuclei of the tissue were counterstained with DAPI and visualized by fluorescence microscopy. The liver sections were washed thrice with PBS, and the images were captured under fluorescence microscope.

### 3,3’-Diaminobenzidine staining

Liver sections, fixed with 4% PFA (v/v), were deparaffinized and subsequently incubated in 0.1 M HEPES buffer (pH 7.4) supplemented with 1.0 mg/mL glucose and 1.0 mg/mL 3,3’-diaminobenzidine (DAB) staining for 5 h at 37°C, followed by two consecutive 5-min washes with normal saline. The nuclei of the tissue were counterstained with hematoxylin and the stained depots were captured under microscope.

### 8-OH-dG detection

PFA-fixed liver sections were permeabilized with 0.2% Triton X-100 in PBS for 10 min. Sections were blocked with 10% normal goat serum in PBS for 1 h at room temperature, followed by incubation with Avidin-TRITC (Sigma-Aldrich) diluted 1:200 at 4°C overnight. Three consecutive 5-min washes with PBS were followed by counterstaining with DAPI. The liver sections were washed thrice with PBS, and the images were captured under fluorescence microscope.

### Immunoblot analysis

Conventional immunoblotting procedures were employed to detect the target proteins. In brief, tissues were collected to extract protein using PBS buffer and lysates were cleared by centrifugation at 15,000 × g for 20 min. Total protein concentration was determined by the Bradford assay. Equal amounts of protein were separated on 4–20% SDS/PAGE and the proteins were transferred onto nitrocellulose membranes. The membranes were blocked for 1 h in Tris buffered saline (TBS) solution containing 5% bovine serum albumin (BSA) and 0.1% Tween-20 and probed with primary antibodies overnight in 0.5% BSA, 0.1% Tween-20, and 0.2% sodium azide in TBS. After washing, the membranes were incubated for 1 h with horseradish peroxidase-linked secondary antibodies (Sigma-Aldrich) in TBS solution containing 0.5% BSA, 0.1% Tween-20, and 0.2% thimerosal. Finally, after three 10-min washes in 0.1% TBS/Tween-20, proteins were visualized by chemiluminometer (LAS 4000, General Electric, Boston, MA, USA). Band intensities were quantified using the *ImageJ* software.

### HPLC and MS analysis

HPLC analysis was performed on an Agilent 1260 series system (Agilent, Santa Clara, CA, USA) with an auto-sampler, binary pump, degasser, and diode array detector. The sample of 10 μL infection volume was separated on Shiseido CapCell PAK C18 column (5μm, 4.6 mm I.D. × 150 mm) with flow rate of 0.6 mL/min. The mobile phase for separation of sample consisted of water (solvent A) and acetonitrile (solvent B) containing 0.1% formic acid each. The gradient elution: 10% of B at 0–3 min, 10–25% of B at 3–25 min, 25–95% of B at 25–30 min. Mass spectrometer was performed on Agilent 6530 Q-TOF mass spectrometer (Agilent, Santa Clara, CA, USA). All acquisition parameters are customized with MassHunter Workstation software LC/MS Data Acquisition. The electrospray ionization (ESI) interface was adjusted positive and negative mode, and mass detection range was *m/z* 50–1700.

The quantitative analysis was performed on Waters 2695 (Waters, Milford, MA, USA) linked with Waters 996 Photodiode Array Detector (PAD). The condition of analysis is same as above HPLC system.

### Statistical analysis

Data were expressed as the mean ± standard error of means (SEM). All data were analyzed by the Student *t* test and a *P* value of 0.05 or less was considered statistically significant (SAS 9.3 version; SAS Institute Inc., Cary, NC, USA).

## Results

### RUE decreases alcohol-induced liver damages

**Fig 1:**
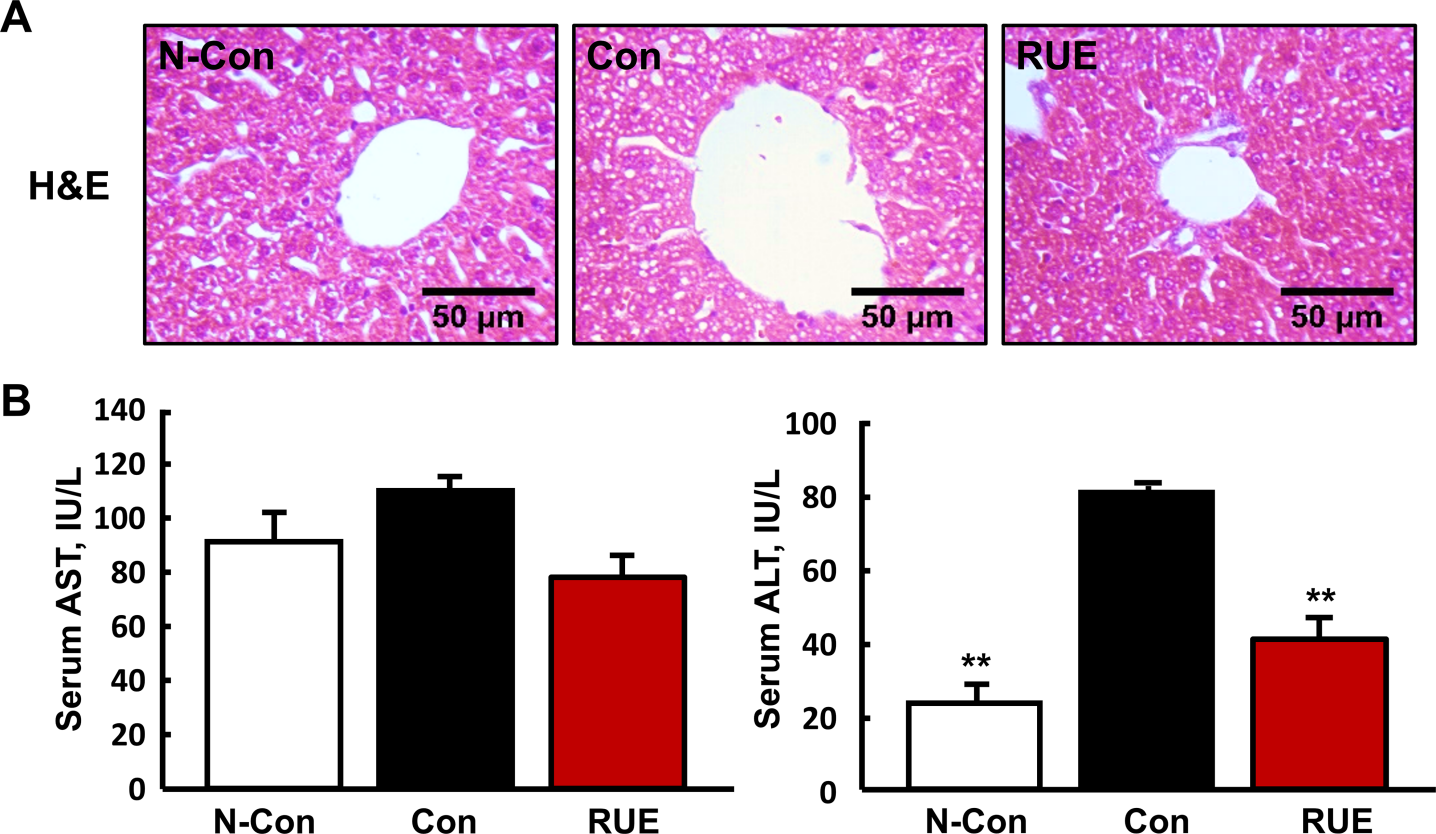
RUE decreases the level of alcohol-induced liver damage markers. (A) Representative images of hematoxylin & eosin (H&E) staining of liver (200× magnification); (B) Levels of aspartate aminotransferase (AST) and alanine aminotransferase (ALT) in serum. All data are presented as the mean ± SEM. (**P < 0.01 vs. control).

Alcohol-induced liver injury was assessed by analyzing hepatic lesions and measuring serum AST and ALT levels. The hepatic lesions were evaluated by H&E staining, but no noticeable alcohol-induced hepatic lesions was observed among the three groups (Fig 1A). On the other hand, the levels of AST and ALT, representative markers for the liver damage, were increased by alcohol consumption, and these were reversed by RUE treatment (Fig 1B). Of note, the ALT level was significantly reduced in the RUE group comparable to that seen in the negative control group.

### RUE reduces alcohol-induced hepatic steatosis by modulating lipid metabolism related-gene expression

**Fig 2:**
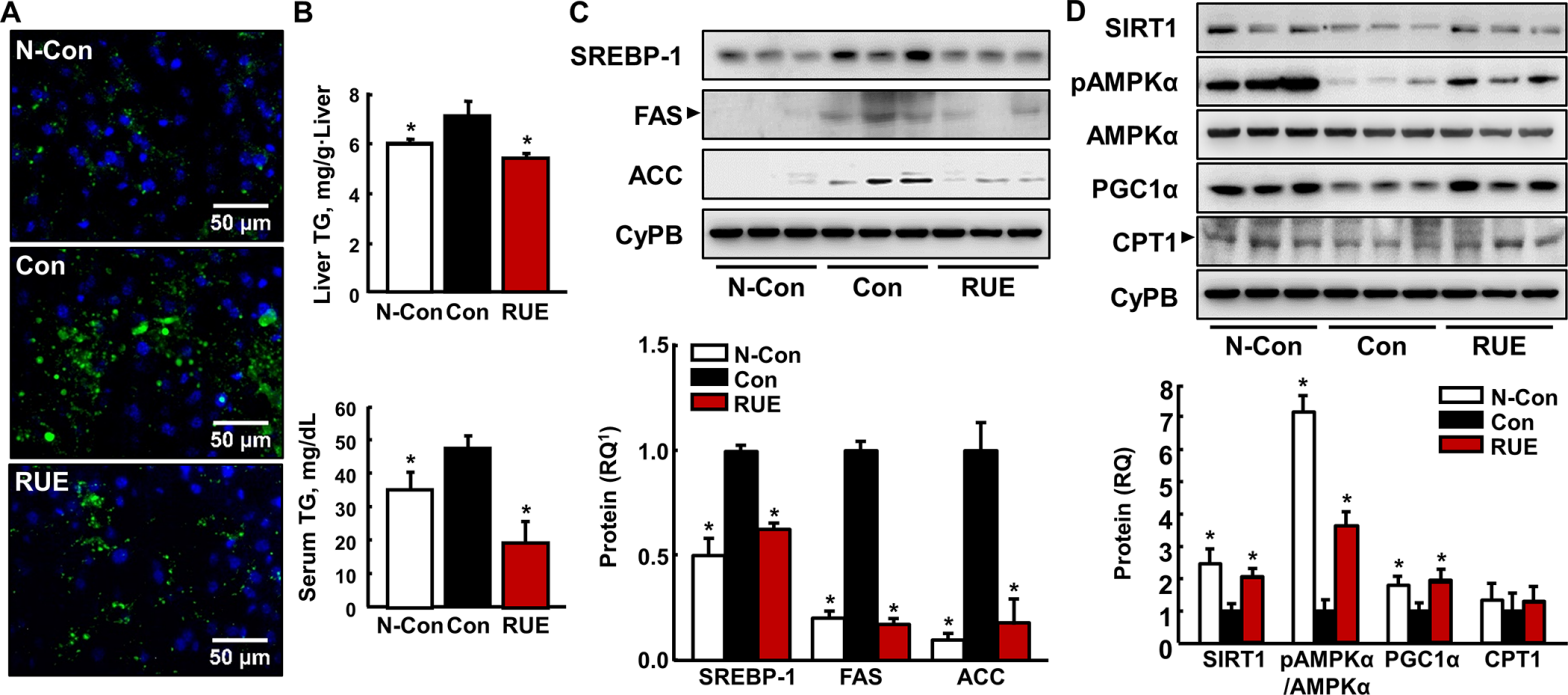
RUE attenuates alcohol-induced hepatic steatosis. (A) Representative images of boron-dipyrromethene (BODIPY) staining of lipid droplets in the liver tissue (200× magnification); (B) Levels of triglyceride (TG) accumulation in serum and liver tissue. All data are presented as the mean ± SEM. (*P < 0.05 vs. control); (C) Immunoblot analyses of lipogenic genes (SREBP-1, ACC, and FAS) in liver tissue with the loading control CyPB. All data are presented as the mean ± SEM. (*P < 0.05 vs. control); (D) Immunoblot analyses of fatty acid oxidative genes (SIRT1, AMPK-α, PGC1-α, and CPT1) with the loading control CyPB. All data are presented as the mean ± SEM. (*P < 0.05 vs. control) ^1^RQ, relative quantity.

The effects of RUE on hepatic lipid accumulation were assessed by boron-dipyrromethene (BODIPY) staining. In the BODIPY tissue staining analysis, RUE decreased the amount of lipid droplets in the liver elevated by alcohol treatment (Fig 2A). In addition, the RUE treatment significantly reduced alcohol-induced TG accumulation in serum and liver tissue (Fig 2B). Subsequently, the expressions of proteins involved in the regulation of lipid synthesis and oxidation were measured by immunoblot analysis. We found that RUE significantly inhibited the expression of lipogenic proteins such as SREBP-1, FAS, and ACC (Fig 2C). Conversely, the expression and activity of SIRT1, AMPKα, and PGC1α, key proteins involved in fatty acid oxidation, was upregulated compared with that in the control group (Fig 2D). However, there was no statistical difference in CPT1 expression level between control and RUE groups.

### RUE protects against alcohol-mediated hepatic inflammation by inhibiting MAPK/NF-kB pathway

**Fig 3:**
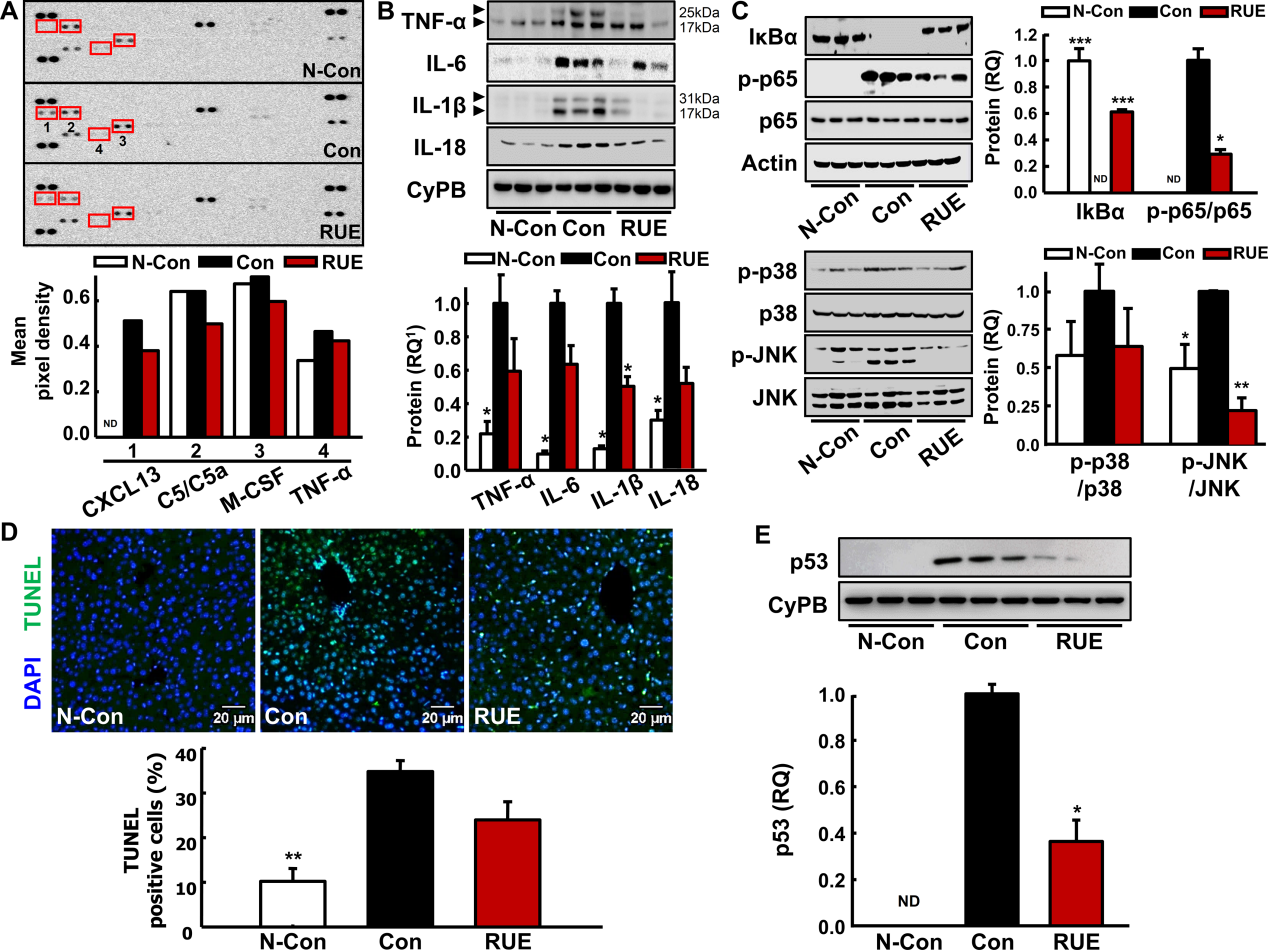
RUE decreases expression of inflammatory cytokines through MAPK-NF-κB pathway. (A) Inflammatory cytokines levels in serum were analyzed using mouse cytokine antibody array. A graph depicting the mean pixel density of dot blots; (B) Immunoblot analyses of inflammatory cytokines (TNF-α, IL-6, IL-1β, and IL-18) in the liver tissue with the loading control CyPB. All data are presented as the mean ± SEM. (*P < 0.05 vs. control); (C) Immunoblot analyses of p38 and JNK MAPK-NF-κB inflammatory pathway. Actin, non-phosphorylated p65, p38, and JNK were examined as the loading controls, and a graph depicting the quantification of the relative abundance of the genes is shown. All data are presented as the mean ± SEM. (*P < 0.05; **P<0.01; ***P<0.001 vs. control). Not detected bands were indicated as ND in the quantifications; (D) Representative images of terminal deoxynucleotidyl transferase dUTP nick end labeling (TUNEL) assay in the liver tissue (200× magnification). TUNEL-positive cells were detected by immunofluorescence analysis. Histograms represent the quantification of fluorescein isothiocyanate-labeled nucleus over the total number of nucleus. Data are presented as the mean ± SEM. **P<0.01 vs. control); (E) Immunoblot analysis of p53 with the loading control CyPB. Data are presented as the mean ± SEM. (*P < 0.05 vs. control). ^1^RQ, relative quantity.

The levels of serum inflammatory cytokines were analyzed using the mouse cytokine array. The expression of cytokines related to the activation and migration of macrophage (CXCL13, C5/C5a, M-CSF, and TNF-α) were significantly attenuated by the RUE treatment (Fig 3A). In parallel with these findings, our subsequent analysis showed the trend of decrease in hepatic inflammatory cytokines by the RUE treatment, including TNF-α, IL-6, IL-1β, and IL-18 (Fig 3B). Especially, the expression of IL-1β was significantly downregulated by RUE treatment. Although there was no statistically significant changes in anti-inflammatory effect based on the contents of the other cytokines by the RUE treatment, the trend of decreases in TNF-α, IL-6, and IL-18 was observed. Representing signaling pathways for the inflammation triggered by alcohol treatment, alcohol-induced phosphorylation and activation of NF-κB p65, and activation of JNK MAPK were significantly inhibited in the RUE-treated group (Fig 3C).

We further examined the levels of hepatic apoptosis by TUNEL assay to investigate whether RUE prevents apoptosis of hepatocytes due to alcohol-induced liver cell damage. Apoptotic cells significantly increased in liver tissues of control group. Although there was no statistical significance in TUNEL assay between control and RUE groups (Fig 3D), it is likely that the apoptotic signaling was potentially modulated by RUE treatment. In particular, the activation of p53, which was induced by alcohol consumption, was remarkably downregulated by the RUE treatment (Fig 3E).

### RUE decreases alcohol-induced oxidative liver damage by ROS scavenging

**Fig 4:**
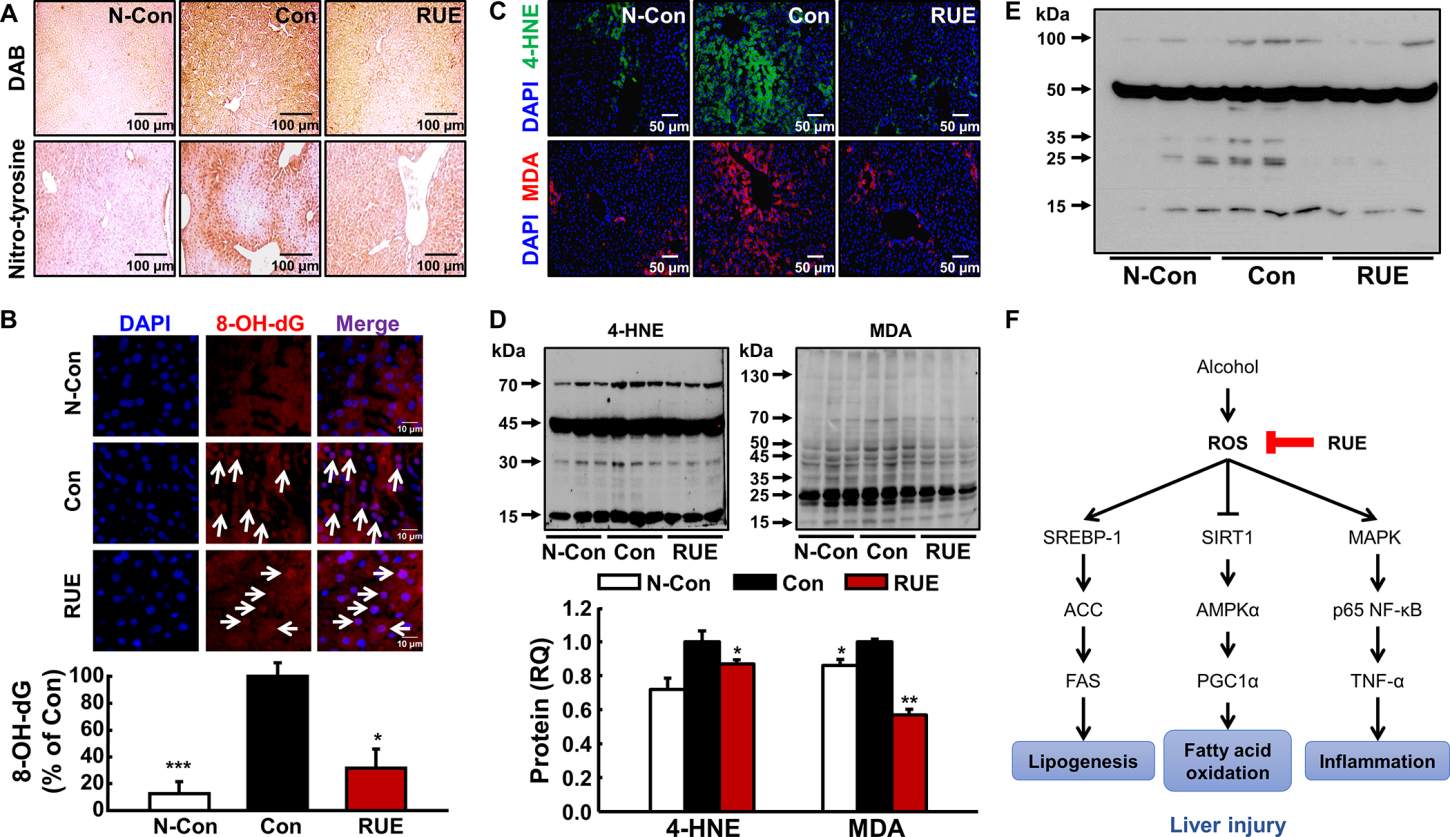
RUE attenuates alcohol-induced oxidative damages through its antioxidant activity and proposed molecular pathway in liver. (A) Representative images of 3,3’-diaminobenzidine (DAB) and nitro-tyrosine in the liver tissue (40× magnification). DAB and nitro-tyrosine were used to measure the level of reactive oxygen species (ROS) and reactive nitrogen species, respectively; (B) Representative images of DNA damages measured by Avidin-TRITC staining in the liver tissue (250× magnification). Avidin-TRITC-stained nucleus can be recognized as 8-OH-dG and nucleus were stained with 4’,6-diamino-2-phenylindole (DAPI). Histograms represent the quantification of avidin stained nucleus over the total number of nucleus. Data are presented as the mean ± SEM. (*P < 0.05; ***P<0.001 vs. control); (C, D) Representative images of immunohistochemical and immunoblot analyses of lipid peroxidation adducts, 4-hydroxynonenal (4-HNE) and malondialdehyde (MDA) (40× magnification). A graph depicting the quantification of the relative abundance of the genes is shown. Data are presented as the mean ± SEM. (*P < 0.05; **P<0.01 vs. control); (E) Immunoblot analysis of glutathione disulfide (GSSG); (F) Proposed molecular pathways for the preventive effect of RUE against alcohol-induced liver injury through ROS scavenging.

To determine the relationship between the antioxidant capacity of RUE and prevention of alcohol-induced liver injury, the levels of ROS and reactive nitrogen species (RNS) were assessed in the liver tissue by DAB staining and nitro-tyrosine staining, respectively. The RUE treatment notably reduced DAB and nitro-tyrosine-positive signals compared with those in the control group (Fig 4A), which indicated that RUE scavenged hepatic ROS and RNS, which were induced by alcohol treatment. In addition, oxidative DNA and lipid damages were evaluated by measuring the levels of Avidin-TRITC (8-OH-dG) and lipid peroxidation adducts [4-hydroxynonenal (4-HNE), and malondialdehyde (MDA)], respectively. In particular, alcohol-induced 8-OH-dG formation was attenuated by the RUE treatment (Fig 4B); furthermore, 4-HNE and MDA production, the surrogate markers of lipid peroxidation, were significantly reduced by the RUE treatment (Fig 4C and 4D). Finally, hepatic glutathione disulfide (GSSG) level was measured using anti-GSSG antibody to determine the protective effects of RUE on hepatic redox status. The increase in GSSG level by alcohol treatment was remarkably reversed by the RUE treatment (Fig 4E).

### Analysis of eight major catechins in the RUE

The RUE and standard mixture of eight major catechins (i.e., Caffeine, (-)-Epigallocatechin 3-gallate, Catechin, (-)-Epicatechin, (-)-Epicatechin 3-gallate, (-)-Gallocatechin, (-)-Gallocatechin 3-gallate, and (-)-Catechin 3-gallate) were analyzed using HPLC and LC/MS to identify the catechins in the RUE via comparison of retention times and MS spectrums. Among the eight catechins, only catechin was identified in the RUE (S1A Fig). In subsequent, the catechin in the RUE was quantified by HPLC analysis (S1B Fig). The amount of the catechin in the RUE was 4.386 μg/mg.

## Discussion

ROS negatively orchestrates global mechanisms involved in various types of liver diseases, e.g., alcoholic liver disease [19], non-alcoholic fatty liver disease [20], hepatitis C virus [21], and acetaminophen-induced liver injury [22]. Thus, excessive production of ROS is the most dominant etiological aspect in alcohol-induced liver injury. To be specific, increased production of ROS induces oxidative damages such as lipid peroxidation and DNA damage, which has further implications. It was reported that lipid peroxidation adducts, such as 4-HNE and MDA, decrease TG export in hepatic cells, deplete GSH, and upregulate TNF-α and TGF-β [reviewed in [23]]. In our study, RUE effectively reduced oxidative damages, as evidenced by the number of 8-OH-dG-positive cells; moreover, surrogate markers of lipid peroxidation (4-HNE and MDA) decreased in the liver tissue section, indicating that RUE may prevent alcohol-induced lipid metabolism disruption by reducing oxidative damages. Considering the close relationship between oxidative stress and hepatic lipid metabolism, we speculated that RUE had an effect on alcohol-induced hepatic steatosis onset.

Previous findings suggested that hepatic SIRT1 plays a pivotal role in lipid metabolism. In particular, alcohol consumption disrupts hepatic SIRT1, which results in hepatic lipid accumulation [5]. SIRT1 disruption inhibits the activation of AMPK-α [24] and subsequently decreases the expression of PGC1-α, resulting in the reduction of fatty acid oxidation [25]. In addition, the decreased activation of AMPK-α increases the expression of SREBP-1, a key regulator of *de novo* lipogenesis as well as other downstream lipogenic enzymes [e.g., ACC and FAS; [26, 27]]. Furthermore, the upregulated expression of ACC and FAS prevents fatty acid oxidation by inhibiting mitochondrial CPT1 [28]. In our study, the hepatic lipid accumulation was significantly reduced by the RUE treatment, which may be attributable to the simultaneous effect of RUE on lipogenic genes (SREBP-1, ACC, and FAS) and fatty acid oxidation-related genes (SIRT1, AMPKα, and PGC1α), respectively (as shown in the Fig 2C and 2D).

Alcohol-induced oxidative stress primarily leads to hepatic steatosis, and the steatosis can be deteriorated by stimulating inflammatory signaling pathways, e.g., JNK MAPK [6, 29], NF-κB [30], or inducing the release of pro-inflammatory cytokines [30]. In particular, alcohol-induced ROS activates NF-κB signaling pathway [31] which, in turn, stimulates TNF-α production [32] that is associated with progression of alcoholic hepatitis. Other studies also demonstrated that the alcohol-induced suppression of cAMP disinhibits NF-κB signaling axis, thereby enhancing TNF-α transcription [33]. These findings strongly suggest that blocking ROS can be a key preventive process against the early event of alcoholic liver injury through the control of the inflammatory condition of the liver caused by excessive alcohol consumption. Interestingly, we found that the activation of MAPKs (p38, and JNK) and NF-κB p65, which was likely induced by alcohol-induced ROS accumulation, was significantly inhibited by the RUE treatment. Furthermore, TNF-α expression was downregulated by the RUE treatment, confirming the suppression of NF-κB signaling cascade. Moreover, the hepatic expression of IL-6, IL-1β, and IL-18, the downstream targets of NF-κB were downregulated in the RUE group. Finally, alcohol-induced decrease in AMPK activation was significantly restored in the RUE group, indirectly indicating that RUE prevents the alcohol-induced suppression of cAMP level in the liver.

In addition to the alcohol-induced inflammation in the liver, hepatic apoptosis is one of the major hallmarks of alcohol-induced pathological phenotypes of liver [reviewed in [34]] and the MAPK–p53 axis is a major signaling pathway involved in the apoptosis of hepatocytes. Specifically, the activation of p38 MAPK in bovine hepatocytes induces p53 transcriptional activation and translocation to the nucleus [35]. Similarly, activation of p38 MAPK, resulting in elevated cellular ROS production, regulates p53-mediated apoptosis in the human hepatocytes [36]. In the current study, the RUE decreased MAPK activation and p53 induction. Subsequently, there was a trend of decrease in the number of TUNEL-positive apoptotic cells in the RUE group (although the difference was not statistically significant; *P* > 0.05). Such marginal effects may have been contributed by the experimental design (e.g., relatively short feeding period), suggesting that RUE may significantly affect hepatic apoptosis in chronic alcohol consumption model.

Collectively, oxidative damage, induced by excessive accumulation of ROS, is a major effector in alcohol-induced liver injury. Thus, plants or fruits rich in antioxidants could be effective in preventing such burdens in the liver [37, 38]. Many studies have investigated the protective potency of antioxidative constituents in natural plants, particularly phenolic compounds, against alcoholic liver injury in diverse models [39–41]. Previous findings suggested that RUE is a promising antioxidant source [12, 13] and in the parallel with these studies, we demonstrated that the antioxidant capacity of RUE was compatible to that of vitamin C (EC_50_ of vitamin C and RUE was approximately 10 mg/100 mL and 11 mg/100 mL, respectively; data not shown). Considering that vitamin C is used as a reference standard flavonoid to assess antioxidant capacity of unknown substance, the antioxidant capacity of RUE is noteworthy. Therefore, the substantial antioxidative capacity of RUE may be responsible for various hepatoprotective mechanisms (e.g., lipogenesis, fatty acid oxidation, and NF-κB–TNF-α axis-mediated inflammatory pathway; Fig 4F).

Detailed studies on chemical characterization demonstrated polyphenolic catechins as major bioactives of the RUE [16, 42, 43]. Accordingly, we analyzed the eight major catechins in the RUE using HPLC and LC/MS (S1 Fig), and we could detect only catechin that is reported as one of major bioactive antioxidant compounds in the RUE [16, 42, 43]. In addition, the catechin compound has been reported as a bioactive compound with antioxidant effect and thus protective against alcohol-induced liver injury in rats through suppression of ROS-mediated NF-kB signaling pathways [44]. It provides the possibility that the catechin would provide, in part, the crucial contribution to the protective effects of RUE on the alcohol-induced liver injuries such as hepatic steatosis and inflammation. However, further investigations are warranted to identify major constituents in the RUE, which are responsible for the protection against the liver injury and examine clinical implications of RUE in alcoholic patients to explore its potential as a hepatoprotective agent. Taken together, we here demonstrated casual relationships of antioxidant capacity of RUE with alcohol-induced liver injury in mice. Our study was carried out in the perspectives of suppression of lipogenesis in the liver tissue, partly via repression of FAS, as well as inhibition of hepatic inflammation by modulation of NF-κB signaling axis. Thus, the current study provides evidence for a novel functional role of RUE against alcohol-induced hepatic steatosis onset and its molecular mechanisms.

## Supporting information

S1 Fig Identification and quantification of major catechins in the RUE by HPLC and LC/MS analysis. (A) LC/MS spectrums of standard catechins and catechins in the RUE; (B) UV chromatogram of the standard catechins and that of the RUE. The peak area were used to quantify the amount of catechin in the RUE.
(TIF)

S1 File Unadjusted blotting bands. Blotting band images from immunoblot analyses were detected by LAS 4000 (Chemiluminometer, General Electric).
(DOCX)
