# Supplementary figures and images for: Root bark of Ulmus davidiana var. japonica restrains acute alcohol-induced hepatic steatosis onset in mice by inhibiting ROS accumulation
Source: PLoS One. 2017 Nov 27;12(11):e0188381. doi: 10.1371/journal.pone.0188381 (PMC5703503; doi:10.1371/journal.pone.0188381)

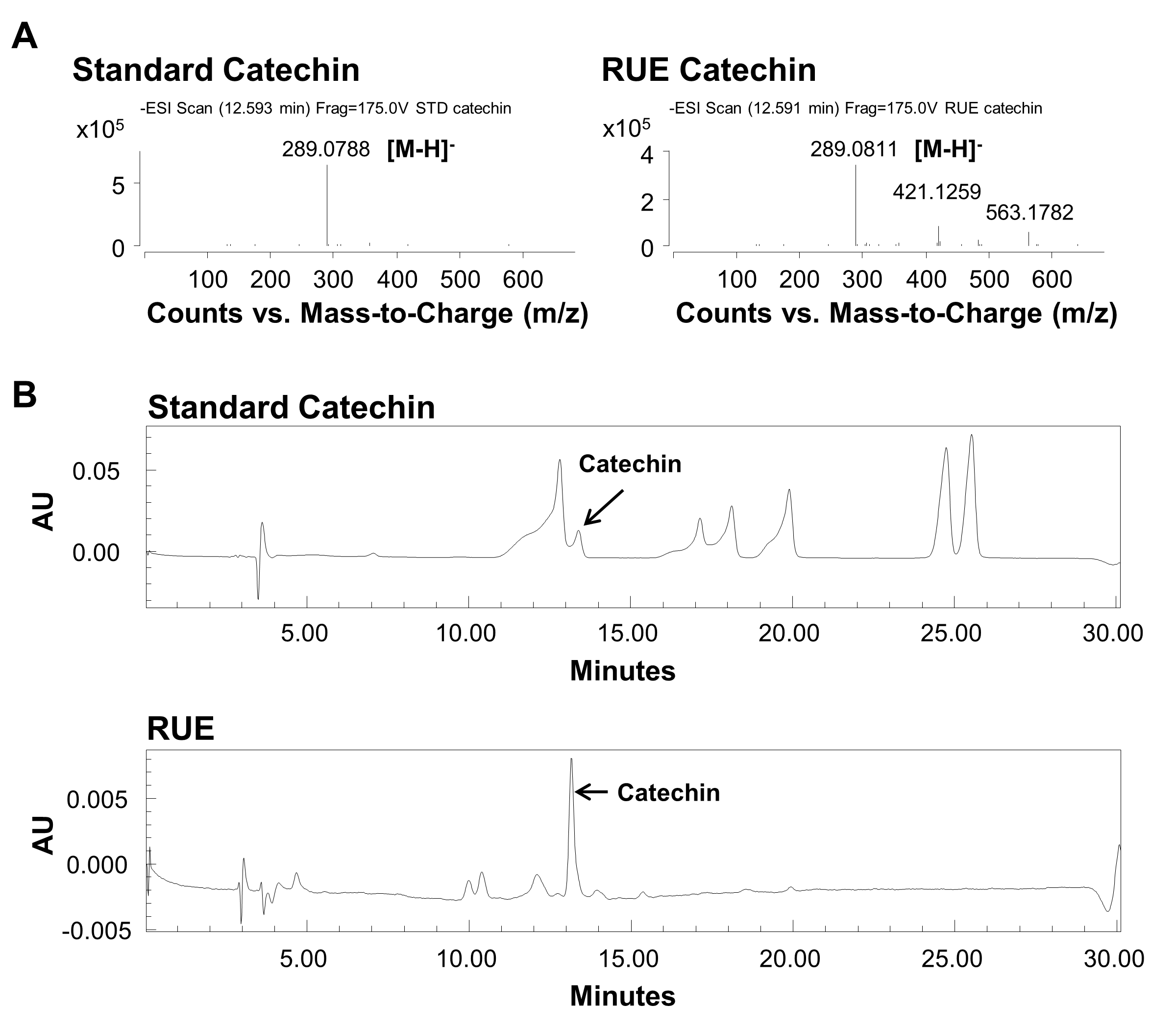

Supplement: S1 Fig — (A) LC/MS spectrums of standard catechins and catechins in the RUE; (B) UV chromatogram of the standard catechins and that of the RUE. The peak area were used to quantify the amount of catechin in the RUE. (TIF) [file pone.0188381.s001.tif]
