# Supplementary material for: Root bark of Ulmus davidiana var. japonica restrains acute alcohol-induced hepatic steatosis onset in mice by inhibiting ROS accumulation
Source: PLoS One. 2017 Nov 27;12(11):e0188381. doi: 10.1371/journal.pone.0188381 (PMC5703503; doi:10.1371/journal.pone.0188381)
Supplement: S1 File — Blotting band images from immunoblot analyses were detected by LAS 4000 (Chemiluminometer, General Electric). (DOCX) [file pone.0188381.s002.docx]

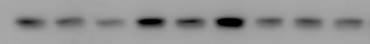

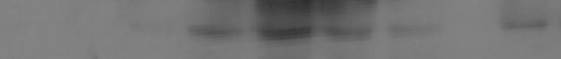

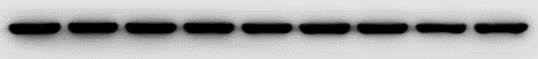

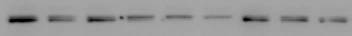

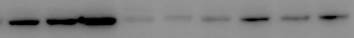

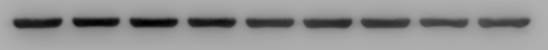

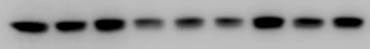

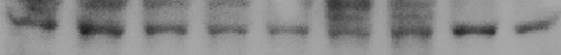

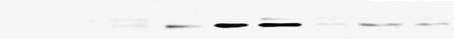


**ACC**

**SREBP-1**

**FAS**

**SIRT1**

**pAMPKα**

**AMPKα**

**PGC1α**

**CPT1**

**CyPB**

**Con**

**RUE**

**N-Con**


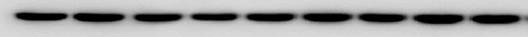

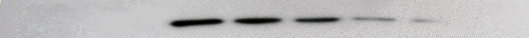

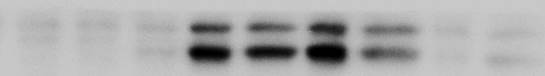

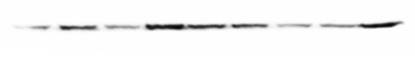

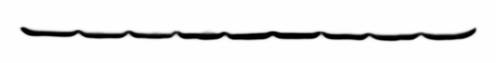

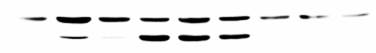


**IL-1β**

**p-p38**

**p38**

**p-JNK**

**CyPB**

**IL-6**


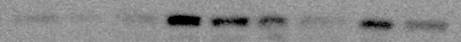


**IL-18**


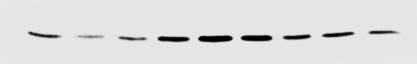


**IκB**


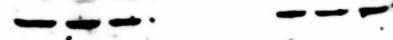


**p-p65**


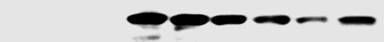


**p65**


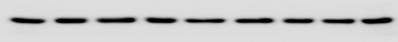


**JNK**


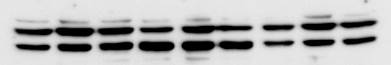


**p53**

**TNF-α**


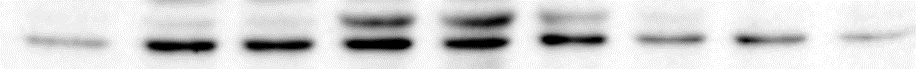


**Con**

**RUE**

**N-Con**


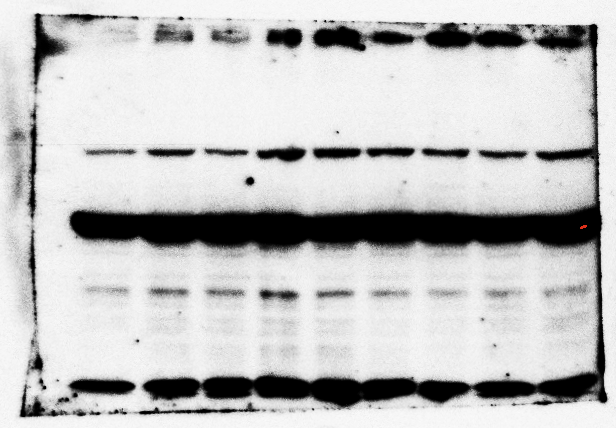

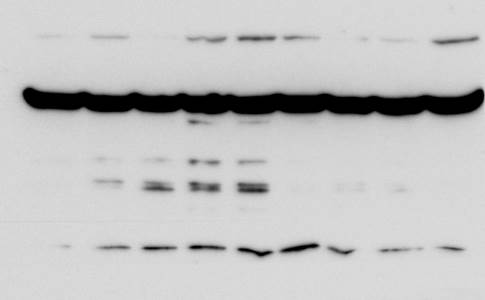


**GSSG**

**4-HNE**


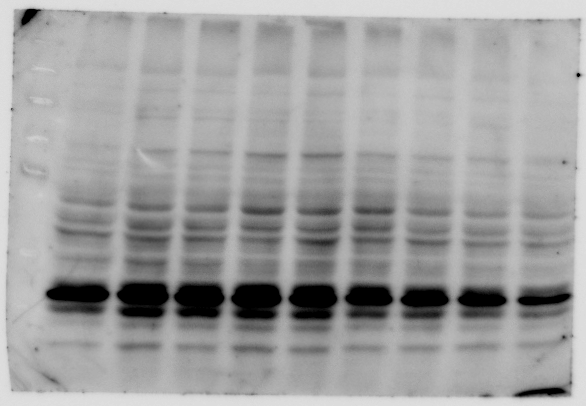


**MDA**

**Con**

**RUE**

**N-Con**
